# Cocreation of a Digital Tool for Proactive End-of-Life Communication: Protocol for a Participatory Action Research Project

**DOI:** 10.2196/88452

**Published:** 2026-02-25

**Authors:** Malin Eneslätt, Charlèss Dupont, Eva Savolainen, Petter Fjällström, Ida Goliath

**Affiliations:** 1 Department of Health, Education and Technology, Luleå University of Technology, Universitetsvägen 16, Luleå, Norrbotten, 97187, Sweden, 46 920493924; 2 Department of Learning, Informatics, Management and Ethics, Karolinska Institutet, Stockholm, Sweden; 3 End-of-Life Care Research Group, Vrije Universiteit Brussel, Brussels, Belgium; 4 Department of Community Medicine and Rehabilitation, Umeå University, Umeå, Sweden; 5 Department of Neurobiology, Care Sciences and Society, Karolinska Institutet, Stockholm, Sweden; 6 Stockholm Gerontology Research Center Foundation, Stockholm, Sweden

**Keywords:** advance care planning, digital technology, participatory methods, codevelopment, Go Wish, DöBra

## Abstract

**Background:**

Proactive end-of-life (EoL) conversations can help individuals, their significant others, and health care professionals be better prepared to confront dying and future EoL decision-making. Talking about EoL issues may be unfamiliar and difficult; tools are increasingly used to support such conversations. While using digital tools presents many advantages, the development processes of such are seldom robustly reported. The project outlined here has the overall aim of further developing and investigating promotion of early, proactive EoL conversations by cocreating and testing, together with potential community-based end users, a digital version of an existing tool, the DöBra cards.

**Objective:**

The aim of this paper is to outline the protocol for a funded participatory action research project, cocreating a digital tool for proactive EoL conversations, as well as reporting initial steps taken in the project.

**Methods:**

Project design is overall inspired by participatory action research and contains 2 work packages (WPs). WP A encompasses the iterative cocreation process of adapting the initial prototype into a relevant digital tool for the public in Sweden. WP B explores if and how the digital tool can support potential end users and significant others in proactive conversations about future EoL values and preferences. Digital tool development is inspired by a process map and will be conducted in collaborative groups. Data collection for both WPs includes repeated interviews with cocreation partners and meeting documentation. Data from field testing of the tool will encompass audiotaped think-aloud exercises, researchers’ observations, usability data, and event log data. Analyses will be qualitative, quantitative, and to some extent mixed methods, and may include inductive thematic analyses, longitudinal qualitative analysis, and descriptive and inferential analyses.

**Results:**

Following funding and project start in January 2024, ethical approval was granted by the Swedish Ethical Review Authority. A stepwise recruitment strategy focusing on heterogeneity yielded an advisory group with 16 members from 13 organizations, representing broad segments of society despite some drop-off. The cocreation process, together with the advisory group, has produced a pilot version of the digital tool, which is currently being field tested. Since May 2025, 42 testers have been recruited. Data analysis is pending. The digital tool will be publicly launched in 2026, readily available, free of charge, and for anyone to use. Results will be disseminated at scientific conferences, in peer-reviewed journals, and through popular science communication.

**Conclusions:**

The cocreative process outlined in this protocol has the potential to develop a digital tool for proactive EoL conversations that is broadly used in the public by varied end users. The digital tool can thus reach new groups in society, potentially highlighting death as a natural part of life and reinforcing normalization of EoL conversations.

## Introduction

Digital tools are increasingly being developed as they may have advantages over analog tools. However, digitalization also brings forth hinders and challenges in usability and availability to the public, especially in people with cognitive hinders. Furthermore, development processes of web-based social health care tools are seldom being fully reported. This protocol paper, therefore, brings a positive addition to robust reporting of digitalization processes.

Proactive reflection and discussion of end-of-life (EoL) values and preferences before death is imminent, a process referred to as advance care planning (ACP) [1,2], can help individuals, their significant others (SOs), and health care professionals be better prepared to confront dying and EoL decision-making when that time comes [3]. While such EoL communication has traditionally been seen as the realm of professionals, which is often still the case in Sweden [4-11], there is increasing international recognition of a need for early, proactive, community-based ACP processes [12-16], not least in the wake of the COVID-19 pandemic [17-19].

The body of knowledge on benefits of ACP practice is vast. Systematic reviews [20-22] show that ACP leads to increased quality of life, health care satisfaction, and use of palliative care. ACP has been associated with reduction in futile life-support treatment at the EoL [20,23] and decreased undesirable hospital admissions [20-23]. International studies [24-27] show that large parts of populations, approximately 60% to 90%, are willing to participate in ACP conversations.

Document-based ACP practices have proven controversial [28-30]. Instead, early and proactive ACP approaches—focusing on preparation of individuals, families, and professionals to be able to make the best possible in-the-moment decisions when they later become necessary—are gaining interest [3,13,21,31]. This focus matches the approach to ACP taken in this project, focusing on reflection and conversations on broad EoL issues in the public.

As talking about EoL issues and dying may be unfamiliar and difficult, tools are increasingly used to support such conversations [32,33]. These tools generally aim to facilitate engagement in ACP discussions by supporting individuals and their SOs to reflect on and/or make decisions for future care and treatment with health care professionals [34]. Initiatives to support community-based, non-professionally led ACP conversations on broader EoL topics are less common, but increasingly being developed [14,35,36].

The Swedish DöBra card is a tool to support EoL conversation that was translated and adapted from the US Go Wish card game, developed by palliative care physician Elizabeth Menkin and the nonprofit organization Coda Alliance [37]. Each card contains a statement, based on seminal research [38] on factors considered important at EoL by patients, family, and caregivers. The process of reflecting on, prioritizing, and ranking card statements serves as the basis for a focused discussion on future EoL care goals [39].

Translating and adapting the Go Wish into the DöBra cards was initiated in 2012, in response to the lack of proactive EoL conversations in the Swedish context at the time [40]. This involved an extensive, cocreative process within a project group of community partners. The DöBra cards have been well-received when tested in various groups in Sweden, that is, community-dwelling older adults [40-42]; staff, residents, and family members in residential elder care [43-45]; and among the Indigenous Sámi [46]. The DöBra cards have been publicly available for purchase since 2018 (without any revenues for researchers); our research indicates an appreciated conversation tool that has had an impact in the public [47].

While the original US Go Wish cards are available online on a basic webpage, scientific studies on the digitalization of the Go Wish cards remain scarce. A recent publication reported on the digitalization of a Go Wish-inspired ACP tool [48]. This project takes inspiration from previous work in Belgium, where a digital version of the Flemish Go Wish cards was developed in a larger project that cocreated a dementia-friendly ACP website [49-53].

Web-based tools can have advantages over paper-based or physical tools as they can be accessed online at any time and place, be used at one’s own pace, alone or together with professional or family caregiver over geographical distances, and have potential to reach a wider and more diverse population [54]. Furthermore, web-based tools can include interactive features that allow tailoring to individual needs and preferences [55]. However, limitations highlighted in a systematic review [14] include web-based ACP tools rarely being developed in collaboration with potential end users, that content was not always evidence-based, and tool development was seldom scientifically evaluated. Furthermore, the level of detail in reporting on the development processes varies, and sometimes a clear description of the digital transition is even left out (Dupont et al, unpublished data, February 2026). In this project, the digital ACP tool will be a further adaptation of the robustly researched Swedish DöBra cards [40-47,56], based on the internationally well-studied Go Wish cards [37,39,57-63], to be developed in a cocreative process and evaluated in manners that allow comparison with previous studies [48,49,64].

In this paper, we outline the protocol for a funded participatory action research (PAR) project, as well as report on initial steps taken in the project. The overall aim of the project is to further develop and investigate the promotion of early, proactive EoL conversations by cocreating and testing, together with potential community-based end users, a digital version of an existing ACP conversation tool, the DöBra cards.

## Methods

### Study Design

Overall project design is inspired by PAR, involving (1) a dynamic and cyclic process of problem identification, planning, action, and evaluation which incrementally changes and builds further on lessons learned, based on (2) partnerships between researchers and community-based stakeholders [65].

**Figure 1.**
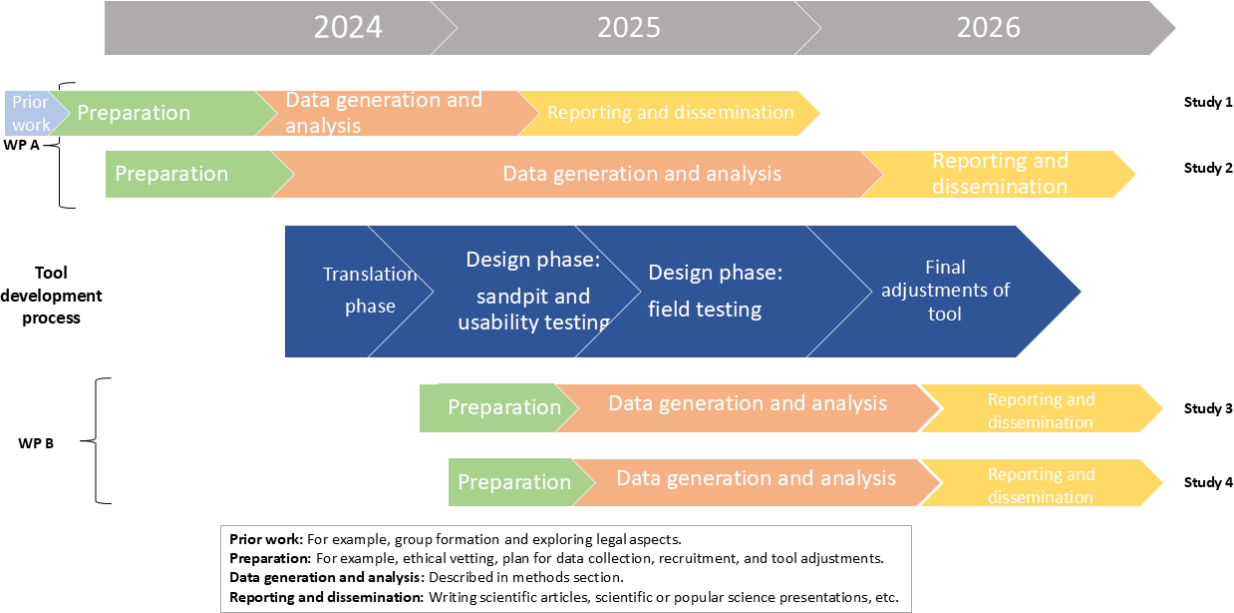
Timeline of tool development and work packages (WPs).

This project consists of 2 work packages (WPs). WP A focuses on the iterative cocreation process of adapting the initial prototype into a relevant digital tool for the public in Sweden. WP B explores if and how the digital tool can support potential end users and SOs in proactive conversations about future EoL values and preferences. Figure 1 presents an outline of the tool development process and WPs.

### Tool Development Process

The process by Elwyn et al [66] for development of web-based decision support interventions inspires the adaptation process with modifications made to enhance our focus on PAR and cocreation. Four intertwined collaborative groups [66] facilitate the process (Textbox 1):

Textbox 1. **Overview of the collaborative groups.**

*Project management group:* A core researcher group, involved in all project phases, responsible for general project management and setting up other groups. The project management group will process feedback from the other groups and make final decisions on content, design, and testing.

*Advisory group:* Various community partners representing potential end users of the tool, recruited via existing and new community contacts, with diverse ages, backgrounds, experience, and competencies. The advisory group gives repeated feedback on the tool’s content, design, usability, and accessibility, primarily in the early design phase.

*Scientific reference group:* Diverse researchers, with expertise from, for example, health sciences, palliative care, health informatics, computer sciences, design, ethics; the scientific reference group will both meet with the advisory group for feedback on adaptations and inform and contribute to research studies.

*Technical production group:* This group will design and build a web-based tool based on input coordinated and communicated by the project management group. The project management group will have a contractual agreement with this group, directly supervising the technical production group.

Based on Belgian experiences [49] and the process map [66], tool development is in phases (Textbox 2). Given the dynamic and cyclic PAR approach, the following is a guiding outline—open for adaptation.

Textbox 2. **Overview of tool development phases.**

*Storyboard step*: All collaborative groups meet for discussions of storyboard version, feedback looped through technical production group and project management group who deliver the next version.

*Sandpit step:* In this phase of exploration and testing new ideas, the advisory group provides ideas and feedback to be looped through the technical production group and project management group before sandpit-testing with approximately 10 potential end users.

*Usability step:* Feedback from sandpit testing is addressed by the project management group and technical production group before discussing the usability version with the advisory group and scientific reference group, resulting in a version for usability testing with approximately 10 new individuals.

*Field testing:* When the prototype is stable with major issues of navigation and structure processed, field testing commences. The prototype will be tested in a heterogeneous sample of approximately 50 individuals, including approximately 30 dyads of target persons and their significant others who will test the digital tool on their own for approximately 6 wk.

*Final tool:* Following adaptations and discussions in all groups after field testing, a final digital DöBra card tool will be launched.

### Recruitment and Participants

In line with the tool development process, recruitment will be conducted in phases. Initially, members of the advisory group were recruited from various community settings, with an emphasis on heterogeneity in experience, background, age, and gender. This recruitment phase is described in detail in the results section. Forming a multiperspective advisory group was important to facilitate cocreation of a user-friendly digital tool, relevant for the broad public in Sweden today. Potential advisory group members were informed of the prolonged engagement as well as the data collection involved with participating in the group.

Subsequent recruitment of participants for the different stages of digital tool testing will be conducted together with the community partners engaged in the advisory group. Given the potentially sensitive nature of reflecting on future EoL preferences, recruitment will be based on active volunteering to minimize risk of perceived intrusion. Researchers will supply oral or written information about the study in various fora, and potential participants will make the active choice of contacting researchers to sign up for the study. Inclusion criteria for testing are willingness and ability to engage in reflection on EoL preferences by trying the digital tool for approximately 6 weeks; having access to and ability to use any kind of screen, for example, smartphone, tablet, or computer; and self-assessed language proficiency in Swedish on a level efficient for use of the tool and engagement in interviews. Individuals deemed by researchers unable to independently supply an informed consent will be excluded from data collection but may test the tool outside of the research study if they wish.

Recruitment for testing will focus on heterogeneity in participants’ age, gender, digital skills, EoL experiences, and educational, cultural, and language backgrounds. While no minimum quotas for subgroups have been decided a priori, in order to maximize the potential to reach different groups, recruitment will be conducted stepwise, mainly through organizations in the advisory group. The stepwise procedure is also helpful in steering recruitment toward characteristics that are not externally visible but can be observed in data collection activities, for example, digital skills and language background.

### Data Collection

#### Work Package A

WP A aims to explore the cocreation and adaptation process of transitioning the paper-based DöBra cards into a digital tool in 2 initial substudies (Table 1). Data will consist of meeting documentations in all phases of cocreation and adaptation, including meeting notes and participant observations of group interaction. All adaptations, including rejected suggestions (from group members and testers), will be carefully documented.

**Table 1.** Overview of substudies, data collection, and analyses.

Substudy: tentative focus	Data sources	Tentative data analysis
1: Exploration of the cocreation process	Meeting documentation Interviews with advisory, project management, and technical production group members	Longitudinal qualitative analysis
2: Description of the adaptations made when transferring from a paper-based to a digital tool	Meeting documentation from cocreation process Testing: Think-aloud exercises, observations, interviews, System Usability Scale	Thematic analysis Descriptive statistics
3: Exploration of test person’s opinions and experiences on if/how the digital tool can facilitate reflection on EoL^a^ preferences	Testing: interviews, event log data, System Usability Scale	Thematic analysis Descriptive statistics, inferential statistical analyses
4: Longitudinal exploration of alignment of test persons’ and significant others’ rankings of EoL preferences	Testing: interviews, rankings of EoL preferences at both time points	Interpretive description Observational, longitudinal comparison of rankings

a EoL: end-of-life.

Advisory, project management, and technical production group members will be interviewed individually and in groups at multiple time points during the process to elicit their experiences and views on the cocreative work. Interviews will probe into, for example, members’ reflections on the current state of the process and pressing issues, collaboration in the group, personal learnings, potential conflicts, and problem-solving.

Data from testing will also be used to describe the adaptation process, for example, audiotaped think-aloud exercises and researchers’ observations. Participants will navigate through each prototype version using a think-aloud method [67], for example, verbalizing their thoughts, impressions, and feelings while engaging with the tool. A researcher will observe and note, for example, participants’ physical cues, task management, difficulties, and comments with minimal interference [67].

Usability data generated using the System Usability Scale (SUS) [68,69] will also be included. The SUS is a widely used, simple, reliable, and validated 10-item scale that measures subjective usability in terms of effectiveness, efficiency, and satisfaction.

During a 6-week field testing period, testers’ usage of the digital tool will be analyzed by event log data. Event logging involves storing 3 variables: (1) unique user ID (who interacted), (2) date and time stamp (when the interaction occurred), and (3) an event name (describing the interaction) [70].

#### Work Package B

WP B aims to explore whether and how the digital tool can support potential end users and SOs in ACP conversations about future EoL values and preferences. To this end, interviews with testers will be conducted before the extended field-testing period and after 6 weeks of testing. In addition to sharing their views of how the digital tool technically worked during testing, testers will also be asked to reflect on the tool’s potential to support reflection and conversations about EoL preferences as well as EoL decision-making.

Field testers can, if preferred, test the digital tool together with a significant other. In those cases, we will further explore how the tool might facilitate their communication on the subject and help align target persons’ (TPs) and SOs perceptions of TPs’ EoL preferences. Data consist of TPs and SOs rankings of TPs’ EoL values and preferences in the digital tool at the first and the follow-up interview, as well as their recorded reflections about their choices. Based on our previous findings [41,42], congruence between TPs and SOs will be assessed by placement in top 10 DöBra card rankings in the digital tool and reasoning about preferences. Both interviews explore participants’ perceptions of how the use of the tool and conversations support preparedness for EoL decision-making.

Event log data may be used to study TPs and SOs patterns of tool use during the trial period to explore, for example, if frequent use of the tool leads to more agreement between TPs and SOs (perception of TPs) EoL preferences.

### Data Analysis

Given the iterative and dynamic PAR process used, interactive forms of data collection, as well as the multitude of data sources, data analyses described here are preliminary and will therefore be described briefly.

Analyses of audiotaped and transcribed interview data and think-aloud exercises, field notes, and participant observations may apply inductive thematic analyses [71], interpretive description [72], and longitudinal qualitative analysis [73,74].

Usability as measured by SUS will be analyzed with descriptive statistics and inferential analyses, in combination with above-mentioned qualitative analyses. While a mean score of greater than 68 is a commonly used benchmark to evaluate usability by SUS [75], these measures will be complemented also by qualitative data to assess usability. Combining multiple data sources in evaluating usability is supported by a recent systematic review [76].

In WP B, event log data [70] will be used to analyze frequency and longevity in participants’ use of the digital tool, to inform mixed methods analysis on participants’ experiences of using the digital tool. By combining event log data and qualitative interview data, we can analyze possible links between stated experiences of using the tool with logged frequency and longevity of tool usage, for example, to see if patterns of usage are related to experience. Mixed methods analysis also includes longitudinal analysis of DöBra card rankings and clustering of event log data, with qualitative data on reasoning about EoL preferences and sense of preparedness in the fourth substudy (Table 1).

In general, analyses will be performed by the first author of the study in close collaboration with the last author but always discussed with the full author team throughout the analysis processes. Reliability of data analyses will be strengthened by triangulation of data sources, methods, and investigators [77], as data from various sources, analyses procedures, and researchers’ competences will be combined in the analyses.

### Ethical Considerations

This project and its WPs have been ethically vetted and approved by the Swedish Ethical Review Authority (2024-03538-01). This project adheres to the Declaration of Helsinki as it involves human research and written informed consent has been obtained from all participants. Furthermore, participants were informed that their privacy and confidentiality are protected by deidentification (pseudonymization) of their data. They were also informed that their data will be stored in safe servers in accordance with Luleå University of Technology’s policy. Participants received no monetary compensation.

Although we are not primarily conducting research with people who are themselves nearing EoL in this project, the substantial literature on and experiences of conducting EoL research suggest that people are often positive to participation, even when nearing the EoL [78]. We are committed to individuals being able to make their own decisions about care and research participation as long as they are able [79]. In order to not be perceived as confrontational for individuals who are not comfortable with the topic of EoL, recruitment will be based on active volunteering.

Furthermore, we are aware of the need for ethical reflection in collaboration with community partners. Discussions and considerations of ethical issues will therefore be integrated intrinsically throughout the project. Community partners may have varied considerations regarding ethical behaviors and issues and may not always agree. Negotiating issues of power, confidentiality, and credibility also demand attention in PAR research, requiring proactive and collective reflection [80,81].

## Results

Following major grant funding, the project commenced in January 2024 and has overall been carried out according to the protocol, even though some amendments have been made given the participatory nature. In this section, we will report on the initial steps conducted in the project.

**Figure 2.**
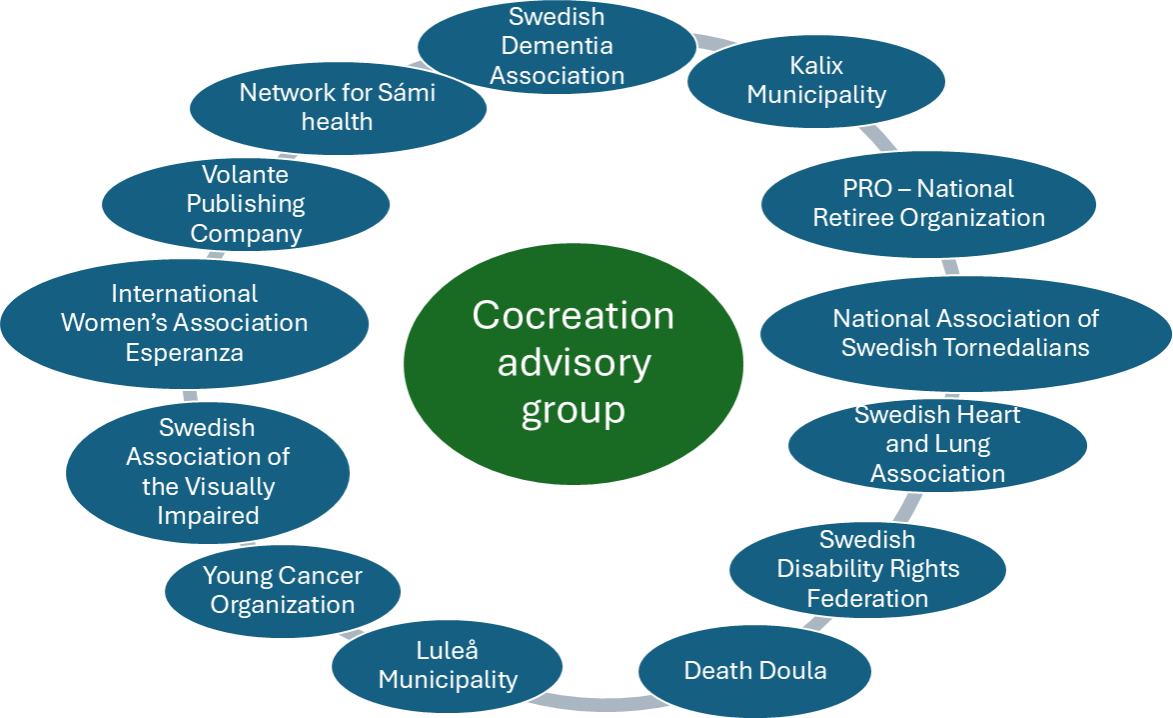
Community partners participating in cocreation in the advisory group. PRO: Pensionärernas Riksorganisation.

Recruitment of community partners involved in cocreation of the tool in the advisory group began in early 2024. A step-wise recruitment strategy was used to avoid recruiting “more of the same.” This strategy resulted in a heterogeneous group (see Figure 2 for an overview of participating organizations). Community partners involved in the previous participatory process translating and adapting the paper-version DöBra cards were first approached, that is, the Swedish Dementia Association and the national retiree organization Pensionärernas riksorganisation; however, involving new representatives from these organizations as the geographical base of the project shifted from the capital area to the northernmost part of the country and as leadership positions had shifted within the organizations. Given that the geographical base of the project constitutes the realm of 2 national minority groups, the Sàmi and the Tornedalians, these groups were approached via their representative organs, that is, the Sàmi parliament and the National Association of Swedish Tornedalians, Tornionlaaksolaiset. In order to gain health care professionals’ input on the project, a registered nurse specialized in dementia care and a nursing aide specialized in education of nursing staff were recruited from nearby municipalities. Striving for heterogeneity in professional input led to recruitment of a death doula, a person offering nonmedical services to dying individuals and their SOs in the final stages of life. Given the ambition to develop a digital tool which would be user-friendly in broad segments of the Swedish public, contact was made with a number of organizations, namely, the Swedish Disability Rights Federation, an organization committed to everybody’s right to self-determination and full participation in society. This is an umbrella organization that wanted to participate with the organizational coordinator as well as 2 representatives from patient organizations: the Swedish Heart and Lung Association and the Swedish Association of the Visually Impaired. These local representatives, in turn, made us aware of the support for older people office in the municipality which works, among other things, with supporting older individuals in managing and using everyday digital platforms. Also, to have perspectives of younger people affected by serious illness, the Young Cancer organization, which works to improve living conditions for young adults living with cancer, was asked to participate. In order to gain perspectives of people not born in Sweden and/or not having Swedish as their first language, the local International Women’s Association Esperanza was approached and asked to participate. Again, with a focus on inclusion and broad perspectives on EoL, a local lesbian, gay, bisexual, transgender/transsexual, queer, and other minority sexual orientations and gender identities organization was approached. Finally, the publishing company selling the paper-version DöBra cards and thus having important insights into how the paper-version cards are being distributed was asked for their interest to participate. All approached parties and individuals initially agreed to participate in the project.

However, during the startup phase, the representative of the lesbian, gay, bisexual, transgender/transsexual, queer, and other minority sexual orientations and gender identities organization and the nursing aide left the advisory group for different personal or organizational reasons. Furthermore, the representatives of the Sàmi Parliament attended the first 2 meetings, but in the second meeting, they communicated a strong wish for the DöBra card statements to be translated and culturally adapted to Sámi settings. This was discussed as a deviating objective from the main project; therefore, the project leader communicated that she envisioned this as a side project that could seek additional funding but not be part of the funded main project. Following this, the Sàmi Parliament representatives left the advisory group. After discussions in subsequent advisory group meetings, the group jointly decided that participation of Sámi representatives was important, and the project leader was given a mandate to try to find other representatives. *The Network for Sámi Health*, an organization focused on promoting health and equitable care for the Sámi people, was therefore approached and agreed to participate.

Some organizations had double representatives in order to cover for each other, making the total number of advisory group members 16: 13 women and 3 men. Not all members have joined every meeting, which has been held in hybrid form after the first physical meeting in Luleå in April 2024. A deviation from the original plan is that the scientific reference group has been less involved in the cocreation of the digital tool. This is both due to the considerable size of the advisory group which already brings very varied perspectives as well as logistical difficulties in scheduling meetings. Instead, the project leader has met scientific reference group members individually on demand, as well as circulated updates on the project and tool development progress approximately twice a year.

Field testing of the digital tool was initiated in May 2025, and at the submission date of this paper, 42 individuals had started testing. While data from the cocreation process is currently under analysis, data from field testings remains to be analyzed, scientific results publications are expected during 2026-27, and public tool dissemination is planned during fall 2026. From the discussions in the advisory group, a vision of the final digital tool being readily available, free of charge and for anyone to use, has crystalized. Discussions of which platform to use for dissemination are still ongoing, with a focus on already existing websites or platforms that can offer a stable host for the tool and managing necessary updates. The long-term governance of the tool is another important question that is under discussion, in order to facilitate broad public dissemination and ensure that the tool will be readily available.

Dissemination of research results will be conducted at international and national scientific conferences and by publishing peer-reviewed scientific articles in journals. Given that this is a PAR project, much attention will also be paid to popular-science communication, for example, public presentations and (written/oral) communication in the community organizations’ preferred channels.

## Discussion

### Initial Findings

This paper outlines the protocol of a PAR project while also reporting initial steps taken in the project. The project has thus far been successful in recruiting a heterogeneous advisory group, representing various perspectives of importance for EoL care and decision-making. The importance of engaging potential end users in designing digital tools is increasingly recognized [76]. Working with PAR stimulates change processes in community partners, while generating new knowledge researching the process. In line with previous findings [43,44,47,82], this project is thus likely to have impact during the cocreative development process and not only at its conclusion, which is 1 motivation for the process-oriented design in WP A. When researching and developing the paper-version DöBra cards, several ripple effects appeared, for example, a strong demand for the cards, leading to them being made publicly available for purchase soon after initial testing and organizational engagement to inform about EoL and APC within their community [47]. Spin-off effects are hoped for also from this project, which is why collaborating organizations have been strategically chosen to represent a broad range of potential end users, facilitating impact in varied community settings. Furthermore, the disseminated digital tool itself has the potential to reach new groups and even broader societal segments. Dissemination and use in varied community settings can highlight death as a natural part of life and reinforce normalization of EoL conversations by reframing ACP as a health-promoting activity involving public education and engagement [49,83].

This project feeds into both active community collaboration in research and making individuals and their SOs active partners in discussing future EoL care, thus potentially strengthening alignment of future EoL care with individuals’ preferences. In Sweden, a major reform of the national health care system is ongoing, aiming at reorganizing and delivering health care close to home based on patients’ needs and conditions. This transition includes designing and managing health care services through discussions with patients to strengthen patient participation [84]. Furthermore, the Swedish National Board of Health and Welfare suggests that eHealth solutions can be used to facilitate and encourage patient participation in care and treatment [85]. The broad DöBra statements, covering physical, social, existential, and practical concerns, may facilitate reflection in individuals and their SOs about support needed from formal caregivers, and which needs can be addressed within their social and family circles. Furthermore, the approaches in this project align with the focus of the new Swedish Social Services Act (SFS 2025:400) on preventive measures and proactiveness. Early and proactive engagement in ACP, as addressed in this project, is one means for individuals' needs to be known to others, and for individuals to be prepared to act as discussion partners when meeting health care providers.

From our previous research with the paper-version DöBra cards, we conclude that they have worked well to elicit individuals’ future EoL values and preferences and stimulate ACP conversations in a range of contexts [40-47,56]. However, 1 issue that community-based ACP initiatives have yet to address is how to document and communicate the preferences discussed in nonprofessionally led ACP conversations. The digital DöBra tool may be a key in both documenting and saving information derived from these conversations for individuals to keep, as well as digitally communicating one’s preferences to someone else, even bridging geographical distance. This project, therefore, also contributes critical new knowledge through addressing the option for individuals to document and communicate EoL preferences resulting from an ACP conversation using the digital DöBra tool.

### Strengths and Limitations

This project responds to limited transparent reporting on digital adaptations. Our detailed documentation of prototype changes, think-aloud testing, and continuous input from a heterogeneous advisory group directly addresses this gap. Moreover, from previous research, we know that challenges commonly arise during such transitions, ranging from difficulties in navigation to issues linked to digital literacy [86-89]. Since these challenges are rarely detailed in published work, transparent reporting becomes crucial for ensuring that others can understand what was done, what worked, and what required adjustment. Transparent reporting is thus essential so that future researchers and developers can build on this work and further strengthen the development of accessible and effective web-based tools.

### Conclusions

The cocreative process outlined in this protocol has potential to develop a digital tool for proactive EoL conversations that is broadly used in the public by varied end users. The project’s focus on inclusion and broad perspectives on EoL issues has strengthened the cocreative process but also brought challenges in managing expectations and demands. The digital tool can reach new groups in society, potentially highlighting death as a natural part of life and reinforcing normalization of EoL conversations.

## Supplementary material

10.2196/88452 Peer Review Report 1

## Abbreviations

ACP: advance care planning
EoL: end-of-life
PAR: participatory action research
SO: significant other
SUS: System Usability Scale
TP: target person
WP: work package
