# Supplementary material for: Cocreation of a Digital Tool for Proactive End-of-Life Communication: Protocol for a Participatory Action Research Project
Source: JMIR Res Protoc. 2026 Feb 25;15:e88452. doi: 10.2196/88452 (PMC12935460; doi:10.2196/88452)
Supplement: Peer Review Report 1 [file resprot-v15-e88452-s001.pdf]

FORTE: Forskningsrådet för hälsa, arbetsliv och välfärd

Beredningsgruppens yttrande

**2023-01235**	**Malin Eneslätt**	**Beredningsgrupp: Etabl H 23**

Utlysningsnamn: Etableringsbidrag 2023

Bidragsform: Etableringsbidrag

Projekttitel (svenska): DöBra-kortleken 2.0: Att undersöka främjande av tidiga, proaktiva samtal inför livets slutskede genom samskapande och testning av ett digitalt samtalsverktyg

Sökt inriktning: Fritt

Bedömning

Generella krav

The project is relevant in relation to Forte's area of funding in addressing wide-spread challenge in society.The applicant describes some relevant aspects from a gender perspective, but there is no theoretically grounded gender perspective in the application. The applicant describes potential ethical challenges and how to address them in the project and a renowned ethicist is connected to the project.

Vetenskaplig kvalitet

The aim of this project is to further develop and investigate the promotion of early, proactive end of life conversations by co-creating and testing, together with potential community-based end-users, a digital version of an existing advance care planning conversation tool, the DöBra cards. The project builds on international research and the applicant's previous PhD thesis, and the aim and research questions are well-described and related to specific work-packages and are motivated in the background information. It builds on research from other national contexts but is still original in adapting this research to a Swedish context.The methodology is fairly complex but described in great detail, including also a description of how different stakeholders will be involved. The methodology seems to be drawing on established methods for development, research on and evaluation of digital tools.
The project has a clear multi-disciplinary perspective and it is described in some detail how these different perspectives will interact.

Samhällsrelevans och nyttiggörande

There is a clear societal relevance, not only because we will all die, but also given the move towards close care in Sweden together with the need to develop e-health solutions given resources constraints and an aging population. The project will, if successful, likely benefit advance care planning for patients at the end of life.

The project has a high degree of collaboration, and apart from involving relevant stakeholders throughout the project, also relates to a well-developed methodology for this.

Genomförbarhet

The applicant has the relevant experience and the work plan specifies in detail what will be done and when, and since the project draws on work already done, it seems realistic.

Etableringsbidragets förväntade betydelse

The project will add to the applicant's competence development, the host institution and the research area.

Sammanvägd bedömning

This is a well-described and realistic project on a relevant topic with potential great societal impact and with a well-developed collaboration with stakeholders.

Förslag till beslut (bevilja, reserv, avslå)

Grant
